# Cysteine mitigates the effect of NaCl salt toxicity in flax (*Linum usitatissimum* L) plants by modulating antioxidant systems

**DOI:** 10.1038/s41598-022-14689-7

**Published:** 2022-07-05

**Authors:** Hebat-Allah A. Hussein, Shifaa O. Alshammari

**Affiliations:** 1 Botany and Microbiology Department, Faculty of Science (Girls Branch), Al-Azhar University, Cairo, 11754 Egypt; 2 Biology Department, College of Science, University of Hafr Al-Batin (UHB), Hafr Al Batin, 31991 Saudi Arabia; 3 Biology Department, University College of Nairiyah, University of Hafr Al-Batin (UHB), Nairiyah, 31991 Saudi Arabia

**Keywords:** Ecology, Physiology, Plant sciences

## Abstract

Agriculture, the main water-consuming factor, faces a global water scarcity crisis. Saline water is an alternative water source, while excess NaCl decreases plant growth and productivity of crops. L-cysteine (Cys) is a promising thiol amino acid in plant growth and development. Flax; *Linum usitatissimum* L. is an economical plant with low salt tolerance. NaCl salt stress at 50 and 100 mM inhibited the growth parameters, the photosynthetic pigments, total soluble sugars, total phenols, and amino nitrogen in flax plants. Salt stress led to a marked rise in proline and lipid peroxidation and altered the protein profile. Foliar application of cysteine at 0.8 and 1.6 mM mitigates the unfriendly effects of NaCl stress on flax plants. Cysteine enhanced the growth traits, photosynthetic pigments, amino nitrogen, total phenols, and new polypeptides in NaCl-stressed plants. However, cysteine declined the total sugars, proline, the activity of peroxidase, and ascorbate peroxidase. The results confirmed that cysteine had reductant properties. Furthermore, it decreased the NaCl oxidative stress and maintained the stability of membranes by lowering lipid peroxidation. Overall, the redox capacity of L-cysteine is the cause behind its potential counteracting of the adverse effects of NaCl toxicity on the growth of flax plants.

## Introduction

Water scarcity is the limiting abiotic factor worldwide in agriculture. The increasing water scarcity will cause a progressive decrease in crop production by 2025^1,2^. So it becomes necessary to preserve water sources and find non-traditional resources such as saline water. On the other side, the composition of saline water may exert different effects on plant growth and crop productivity^3,4^. Usually, saline water stimulates the synthesis of reactive free radicals. The reactive free radicals become toxic because they target proteins, lipids, and nucleic acids and increase their rate of degradation^5^. Many plants induce detoxification mechanisms to mitigate the injury of salt stress. Various enzymes are involved in the harmful radical’s detoxification mechanism. Superoxide dismutase is the first defense enzyme that transforms superoxide into H_2_O_2_^6^. Catalase and different classes of peroxidases scavenged the resulting H_2_O_2_. Salinity had adverse effects on water uptake, nutrient availability, chlorophyll content, photosynthesis, stomatal conductance, and root hydraulic conductance^7–9^.

Flax (*Linum usitatissimum* L.) belongs to *Linaceae* and is grown for fiber, seed, or dual purposes^10^. Linseed oil has enriched with Omega-3, linoleic acid, and oleic acid^11^.

L-cysteine (Cys) is α-amino acid with a thiol group, active in enzymatic reactions. Cysteine is the primary organic amino compound that reduces sulfur bonds in plants^12,13^. The product of L-cysteine metabolism is glutathione, which has contributed to antioxidant mechanisms. Moreover, cysteine derivatives can accumulate mineral ions in *Arabidopsis*^14^. The application of cysteine has confident effects in mitigating the abiotic stress on various plant crops^15^. To manage that, the current work aims to study the influence of L-cysteine on increasing the salt-tolerant property of flax plants.

## Result

### Growth parameters

The results presented in (Table 1) showed different responses in flax plants to the different concentrations of NaCl salinity. NaCl salinity at 50 mM significantly (*P* < 0.05) reduced shoot length and fresh and dry weights of shoot and root per plant, while it had no effects on root length. The reduction increased with increasing the concentration of salinity. The results showed that NaCl at 100 mM reduced shoot length, root length, and fresh and dry weights of shoot and root per plant by 51.20, 46.84, 56.13, 54.10, 81.67, and 62.50%, respectively, compared to the control values. On the other hand, foliar application of cysteine (0.8 and 1.6 mM) considerably increased the vegetative growth criteria of flax plants under normal conditions and saline irrigation.

**Table 1:** Effect of L-cysteine on the growth criteria of the salt-stressed flax plants.

Treatments		Shoot length (cm)	Root length (cm)	Shoot fresh weight (g)	Shoot dry weight (g)	Root fresh weight (g)	Root dry weight (g)
NaCl (mM)	Cysteine (mM)						
0	0.00	55.33^b^	15.67^a^	2.69^b^	0.61^b^	0.60^d^	0.08^abc^
	0.80	53.00^bc^	12.33^bc^	4.99^a^	0.78^a^	1.22^bc^	0.14^a^
	1.60	65.33^a^	12.67^bc^	6.57^a^	0.93^a^	1.78^a^	0.11^ab^
50	0.00	31.00^f^	14.33^ab^	1.85^c^	0.45^cde^	0.32^e^	0.04^c^
	0.80	56.00^b^	13.00^bcd^	3.15^b^	0.48^cde^	0.91^cd^	0.06^b^
	1.60	52.00^bc^	11.00^d^	6.63^a^	0.54^bc^	1.50^ab^	0.08^abc^
100	0.00	27.00^f^	8.33^e^	1.18^d^	0.28^e^	0.11^f^	0.03^c^
	0.80	40.33^e^	12.00^cd^	2.99^b^	0.36^de^	0.63^de^	0.06^b^
	1.60	46.67^cd^	15.00^ab^	3.05^b^	0.46^cde^	0.73^de^	0.07^b^
**LSD at *P* < 0.05**		5.88	4.40	0.70	0.15	0.200	0.054

Under normal conditions, 1.6 mM cysteine improved the shoot length and fresh and dry weights of shoot and root by 18.07, 144.24, 52.46, 196.67, and 37.50% compared to the control plants (Table 1). The interaction between salinity at 50 mM and cysteine at 1.6 mM caused significant (*P* < 0.05) increases; 67.74, 258.38, 20.0, 368.75, and 100% for shoot length, fresh and dry weights of shoot and root per plant, respectively, compared to the untreated salinized plants (Table 1). Moreover, cysteine at 1.6 mM improved shoot length, root length, shoot and root dry weights, and shoot and root fresh weights by 72.85, 80.1, 158.5, 64.3, 563.6, and 133.3%, respectively, in the 100 mM NaCl stressed plants.

### Photosynthetic pigments

**Figure 1:**
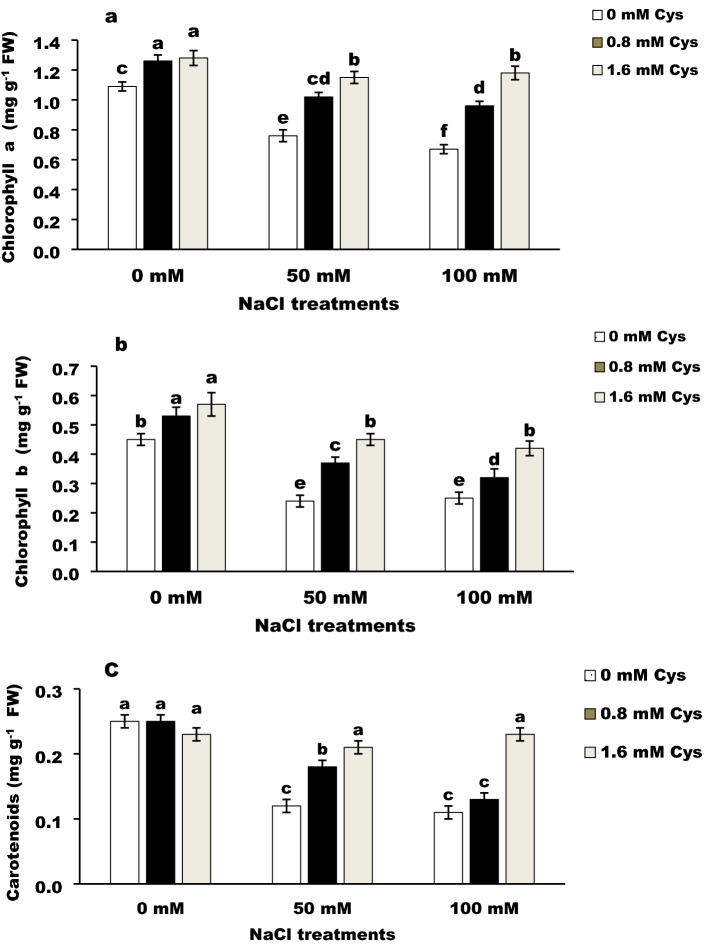
Effect of L-cysteine on photosynthetic pigments; chlorophyll a (**a**), chlorophyll b (**b**), and carotenoids (**b**) in salt-stressed flax plants. Differences are statistically significant at p < 0,05; vertical bars indicate ± SD.

The photosynthetic pigments in the salt-stressed plant are significantly (*P* < 0.05) decreased compared to the control plants (Fig. 1a–c). The maximum decrease reached 38.53%, 46.67%, and 56%, respectively, for chlorophyll (Chl) a, b, and carotenoids in high salt-stressed plants.

Under salinity stress, cysteine at high concentrations increased the Chl a, b, and carotenoids. The increment percentages of chlorophyll a, b and carotenoids were achieved by 1.6 mM cysteine, reaching 51.32, 87.50, and 75.00%, respectively, in low stressed plants and 76.12, 68, and 109.09%, respectively in high stressed ones compared to the corresponding control plants.

### Soluble sugars

**Figure 2:**
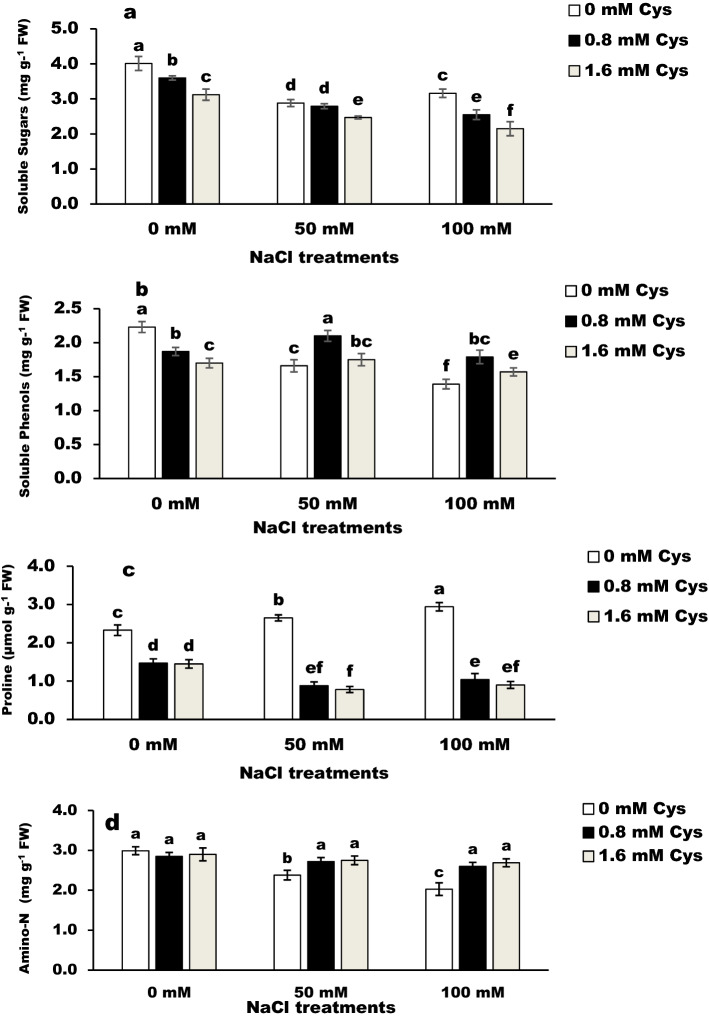
Effect of L-cysteine on soluble sugars (**a**), soluble phenols (**b**) proline (**c**), and amino-N (**d**) in salt-stressed flax plants. Differences are statistically significant at p < 0,05; vertical bars indicate ± SD.

The salt stress significantly (*P* < 0.05) decreased total soluble sugars in flax plants compared to the control plants (Fig. 2a). Similarly, cysteine treatments markedly decreased total soluble sugars in salinized and non-salinized plants compared with their controls.

### Soluble phenols

Phenolic content significantly (*P* < 0.05) decreased by salinity or cysteine when applied separately compared to the corresponding control values (Fig. 2b). While foliar spray of cysteine at 0.8 mM significantly (*P* < 0.05) improved the phenolic contents in salt-stressed flax plants. Moreover, cysteine at 1.6 mM showed no change under the low salinity stress while increasing total phenols under high salt stress compared to the values of non-stressed plants.

### Proline

The proline content was significantly elevated with increasing NaCl concentration (Fig. 2c). However, treatments with cysteine resulted in the opposite effect and led to decreased proline content both in the salt-stressed and non-stressed plants.

### Amino-N

Salinity stress significantly (*P* < 0.05) decreased amino-N in flax plants compared to the control plants (Fig. 2d). However, the amino-N content showed no change in cysteine-treated unstressed plants. The cysteine treatments significantly (*P* < 0.05) increased amino-N content in flax plants under high salt stress compared to the control value.

### The activities of antioxidant enzymes

**Figure 3:**
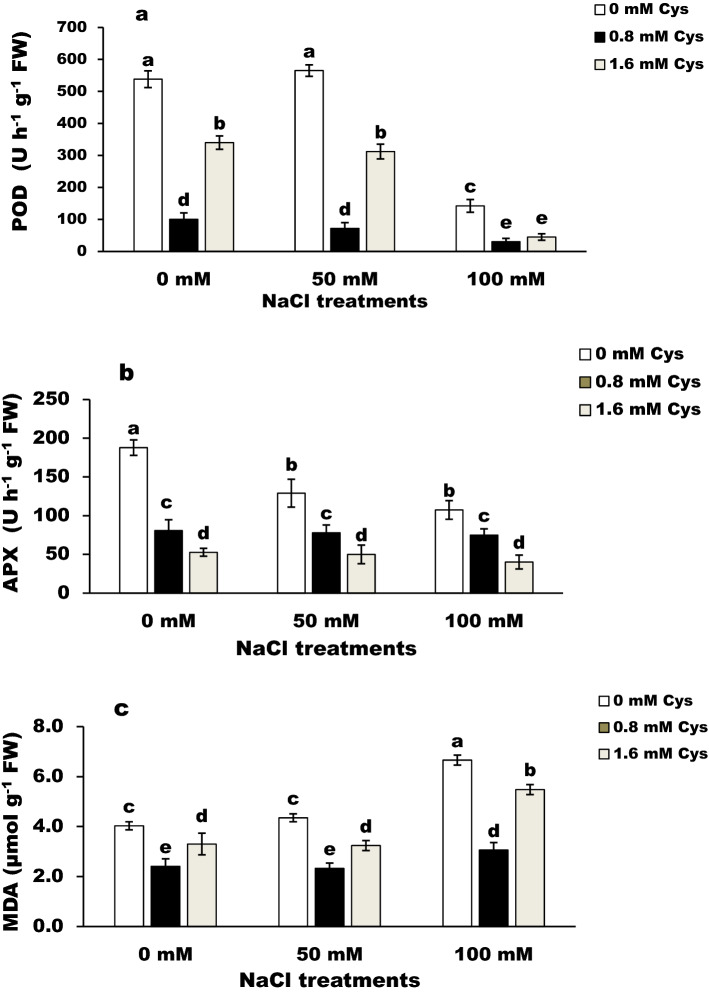
Effect of L-cysteine on the activity of POD (**a**) and APX (**b**) and lipid peroxidation (**c**) in salt-stressed flax plants. Differences are statistically significant at p < 0,05; vertical bars indicate ± SD.

All treatments decreased the activity of peroxidase and ascorbate peroxidase (Fig. 3a,b) enzymes in the leaves of flax plants. The control pants had the highest peroxidase and ascorbate peroxidase activity.

### Lipid peroxidation

Lipid peroxidation, i.e., malondialdehyde (MDA) content, was significantly (*P* < 0.05) increased in salinized plants (Fig. 3c). The highest level of MDA was at a high salinity level. Cysteine treatments, especially at 1.6 mM, caused a significant decrease in MDA in salt-stressed plants compared to their control.

### Protein profile

The results for the protein profile revealed a total of 30 polypeptide bands with different molecular weights ranging from 15 to 180 kDa, which were detected with a polymorphic ratio of 41.9% (Table 2 and see Supplementary Fig. S1 online). Polymorphic polypeptide bands with 180, 110, 93, 73, and 27 kDa were induced in all treated plants. However, bands with molecular weights (66, 50, 46, 40, 37, 35, 32, 29, 23, 20, 18, and 17 kDa) exist in all treatments and are monomorphic bands. Unique polypeptides bands with molecular weights of 100 and 57 kDa are induced in low salt-stressed plants. Furthermore, new polymorphic polypeptides with 120, 62, 34, and 31 kDa, were only at high salinized plants and in all salt-stressed plants treated with cysteine, compared to the other treatments. Interestingly, all salt-stressed plants treated with cysteine exhibited two polymorphic polypeptides with molecular weights of 157 and 43 kDa compared to other treatments. On the other hand, unique bands 64 and 137 kDa appeared with the interaction of 50 mM salinity with a low and high dose of cysteine, respectively.

**Table 2:** Effect of L-cysteine on protein profile in leaves of salt-stressed flax plants.

No	M.W	1	2	3	4	5	6	7	8	9
1	180	**−**	** + **	** + **	** + **	** + **	** + **	** + **	** + **	** + **
2	157	**−**	**−**	**−**	**−**	** + **	** + **	**−**	** + **	** + **
3	137	**−**	**−**	**−**	**−**	**−**	** + **	**−**	**−**	**−**
4	120	**−**	**−**	**−**	**−**	** + **	** + **	** + **	** + **	** + **
5	110	**−**	** + **	** + **	** + **	** + **	** + **	** + **	** + **	** + **
6	100	**−**	**−**	**−**	** + **	**−**	**−**	**−**	**−**	**−**
7	93	**−**	** + **	** + **	** + **	** + **	** + **	** + **	** + **	** + **
8	73	**−**	** + **	** + **	** + **	** + **	** + **	** + **	** + **	** + **
9	66	** + **	** + **	** + **	** + **	** + **	** + **	** + **	** + **	** + **
10	64	**−**	**−**	**−**	**−**	** + **	**−**	**−**	**−**	**−**
11	62	**−**	**−**	**−**	**−**	** + **	** + **	** + **	** + **	** + **
12	60	** + **	** + **	** + **	** + **	** + **	** + **	** + **	** + **	** + **
13	57	**−**	**−**	**−**	** + **	**−**	**−**	**−**	**−**	**−**
14	55	**−**	**−**	**−**	** + **	** + **	** + **	**−**	**−**	**−**
15	50	** + **	** + **	** + **	** + **	** + **	** + **	** + **	** + **	** + **
16	46	** + **	** + **	** + **	** + **	** + **	** + **	** + **	** + **	** + **
17	43	**−**	**−**	**−**	**−**	** + **	** + **	**−**	** + **	** + **
18	40	** + **	** + **	** + **	** + **	** + **	** + **	** + **	** + **	** + **
19	37	** + **	** + **	** + **	** + **	** + **	** + **	** + **	** + **	** + **
20	35	** + **	** + **	** + **	** + **	** + **	** + **	** + **	** + **	** + **
21	34	**−**	**−**	**−**	**−**	** + **	** + **	** + **	** + **	** + **
22	32	** + **	** + **	** + **	** + **	** + **	** + **	** + **	** + **	** + **
23	31	**−**	**−**	**−**	**−**	** + **	** + **	** + **	** + **	** + **
24	29	** + **	** + **	** + **	** + **	** + **	** + **	** + **	** + **	** + **
25	27	**−**	** + **	** + **	** + **	** + **	** + **	** + **	** + **	** + **
26	23	** + **	** + **	** + **	** + **	** + **	** + **	** + **	** + **	** + **
27	20	** + **	** + **	** + **	** + **	** + **	** + **	** + **	** + **	** + **
28	18	** + **	** + **	** + **	** + **	** + **	** + **	** + **	** + **	** + **
29	17	** + **	** + **	** + **	** + **	** + **	** + **	** + **	** + **	** + **
30	15	**−**	**−**	**−**	**−**	** + **	** + **	**−**	** + **	** + **
Total bands		12	17	17	22	26	26	22	25	25
		Monomorphic bands			Unique bands		Polymorphic bands		Polymorphism **%**	
30		12			4		14		46.67%	

Where, M, Protein marker; 1, Control; 2, 0.8 mM Cys; 2, 1.6 mM Cys; 4, 50 mM NaCl; 5, 50 mM NaCl + 0.8 mM Cys; 6, 50 mM NaCl + 1.6 mM Cys; 7, 100 mM NaCl; 8, 100 mM NaCl + 0.8 mM Cys; 9, 100 mM NaCl + 1.6 mM Cys.

## Discussion

Salinity exerts significant constraints on crop production^16^. The salinity stress leads to the imbalance between the free radical production and antioxidant defense systems^17^. Moreover, salinity stress alters the biochemical constituents such as photosynthesis, protein synthesis, and osmoprotectants accumulation^16,18^.

The exogenous L-cysteine alleviated the salinity effects on flax plant growth (Table 1). These results may be due to the converting of L-cysteine into glutathione (GSH), which is essential in plant responses against salinity stress^6,15^. Besides, L-cysteine is involved in protein assimilation; S-nitrosylation during posttranslational modification of proteins, and subsequent plant metabolism. Moreover, it has a regulatory role in energy production and sulfate reduction into organic sulfur^19,20^ leading to improvement of growth parameters and salinity stress tolerance.

The chlorophyll pigments declined markedly in flax plants exposed to salinity stress (Fig. 1). This decline could be due to the damage of chlorophyll through chlorophyllase enzyme, destruction of the chloroplast structure, or the pigment-protein complexes instability^5^. The increment of photosynthetic pigment after foliar application of cysteine is in harmony with^21^. They found that chloroplastic cysteine act as signaling molecules regulating and protecting the photosystems. These results might be due to one of the two probable mechanisms, the first; is the influence of L-cysteine on the antioxidant capacity to mitigate the harmful effects of free radicals generated by salinity stress^17^. The second mechanism is the formation of pyruvate from L-cysteine^22^. Pyruvate subsequently converts into acetyl CoA considers the precursor of many biological molecules like chlorophyll, carotenoids, phytohormones, fatty acids, and proteins. Our results agree and support the second mechanism of the L- cysteine action in alleviating the adverse effects of salt stress on plants.

The salinity stress changed the level of total soluble sugars (TSS) (Fig. 2a). This result may be due to modulating the level of osmolytes such as soluble sugars. The results obtained in the present study are in agreement with the study, which also indicated that modulating and transporting sugars under stress conditions are linked to an increase in lignification and cell wall biosynthesis, as well as to their functioning as osmolytes and antioxidant compounds^23^. In addition, L-cysteine caused lower TSS contents in all treated plants than the control values. This result may be due to the action of cysteine in the reduction of sulfur-donor molecules involved in the biosynthesis of essential organic compounds^12^. Sugars are a precursor for a diverse group of phenolic compounds and are one of the most promising candidates for protecting plants from the adverse effects of environmental stresses^23,24^. Salinity stress reduced total phenols compared to control plants (Fig. 2b). The reduction in phenols levels under salinity stress may be due to its oxidation by the antioxidant enzymes, which withdraw phenols as their substrate. Meanwhile, the increment in the total phenols content of salt-stressed plants in response to L-cysteine may be due to the shifting of total soluble sugars after cysteine treatments. Similarly, Cys significantly increased total phenolic contents in oat plants (var. F-411)^25^. Phenols as substrates for various antioxidant enzymes, increased the plant salinity tolerance and protect the cells from potential oxidative damage and increase the stability of the cell membrane^26^.

Proline acts as a nitrogen-storage, hydrophobic protectant for enzymes and cellular structures, stabilizes membrane, and detoxifies free radicals^19^. The high proline level in salt-stressed plants (Fig. 2c) may be due to the induction in proteolysis or preservation of the precursors of proline^19^. These results agree with^27^. They found that overproduction of proline with increasing the level of salinity stress. Higher proline in plants under salinity stress may be due to its crucial role in maintaining cell turgor or osmotic balance; stabilizing membranes thereby preventing electrolyte leakage, and maintaining reactive oxygen species within normal ranges^28^. On the other side, the lower proline content after cysteine application may be due to its crucial role in detoxifying free radicals salt stress^20^, thus preventing oxidative stress in flax plants.

Amino acids as protective compounds may be included in different biological processes like redox homeostasis against the symptoms of salt stress^29^. Data revealed a lower amino-N content in salt-stressed plants (Fig. 2d). This result may be due to the down-regulation in protein synthesis of salt-stressed flax plants. The higher values of amino-N in salt-stressed plants treated with L-cysteine treatments may be related to the changes in protein metabolism and up-regulating K^+^ transportation to alleviate plants damaged by stress^29–31^. Similarly, it was reported that exogenous application of L- arginine, and L-ornithine had a powerful potential to face the impacts of abiotic stresses on wheat^32^ and sugar beet^6^ plants by promoting the synthesis of amino acids, protein, and antioxidant systems.

All treatments significantly (*P* < 0.05) decreased the APX and POX activities compared to the control values. Salinity may result in an imbalance between the antioxidant enzymes and the reactive free radical. L-cysteine lowered the activities of the antioxidant enzymes. The results are in harmony with^33^. The authors reported a decrease in the activity of CAT, APX, and PPO in Basil plants treated with cysteine at the vegetative stage. To explain the obtained results, we suggested that the mitigating effect of Cys on salt stress might be related to its direct ROS scavenging property rather than its effect on the antioxidant system. This explanation was supported by Cys decreasing the need for activation of the antioxidant system by acting as a ROS scavenger^17^.

Variations in lipid peroxidation content and the activities of antioxidant enzymes explain the influence of cysteine in reducing the ROS level. The high MDA is considered a biomarker of oxidative stress^6^. Salt stress significantly (*P* < 0.05) increased MDA contents in flax plants, while cysteine treatment mitigated this increase. These results proved the influence of cysteine in the alleviation of oxidative damage generated by salt stress. The same result was recorded on the Basil plant by^33^. In the present study, cysteine-treated flax plants take different mechanisms to tolerant salt stress by enhancing non-enzymatic antioxidants; carotenoids, and total phenols. The positive effect of cysteine could be due to the production of glutathione^20,34^ or total phenolic compounds (Fig. 2), which have antioxidant capacity^35^ to scavage excess of free radicals.

Regarding protein profile, the induction of unique polypeptides 100 and 57 kDa at low salinity levels may be due to the presence of 2 responsive genes related to salt stress tolerance. On the other hand, at low salinity levels, unique polypeptides 64 and 137 kDa appeared only in stressed plants treated with low and high concentrations of L-cysteine, respectively. These results mean that L-cysteine may highly affect the expression of 2 different genes depending on its concentration. Moreover, the appearance of 5 new proteins after L-cysteine or salinity treatments predicted their stressor action equally on these five genes. Furthermore, the induction of new polymorphic bands in salt-stressed plants in response to cysteine treatments indicated that L-cysteine has a possible role in vital processes like redox regulation or signal transduction through modulating polypeptides responsible for oxidative tension^19,36^.

## Conclusion

NaCl salt stress negatively impacted the growth parameters and the biochemical constituents of Flax plants. It affects osmolytes, the antioxidant status, and membrane stability. However, L-cysteine enhanced the biosynthesis of photosynthetic pigments, total phenols, amino-N, and new polypeptides. It maintained the stability of membranes by lowering lipid peroxidation and regulated the osmotic balance by adjusting total soluble sugars and proline contents. It also modified the antioxidant status by utilizing the antioxidant enzymes as the defense mechanism to mitigate oxidative salt stress. Ultimately, the multiple positive roles of exogenous cysteine can effectively overcome the adverse effect of NaCl stress on flax growth and development.

## Materials and methods

### Growth conditions

A greenhouse experiment was carried out during the winter season of 2017/2018 at the Faculty of Science (Girl Branch), Al-Azhar University, Nasr City, Cairo, Egypt, to study the effect of salinity (0, 50, 100 mM) and cysteine (0, 0.80, 1.6 mM) on growth and biochemical constituents of flax plants. Flax seeds (Cultivar; Giza 9) were obtained from Agriculture Research Center (ARC), Giza, Egypt. The seeds were sown on November 14th in earthenware pots No. 50 filled with sandy soil. The experiment had a completely random design with six replicates for each treatment. Calcium superphosphate and potassium sulfate were added before sowing. Ammonium nitrate was applied at two intervals after sowing^30,37^.

At 45 days after sowing, three representative samples were taken from each treatment for determining growth traits. Chemical constituents were estimated in the leaf at the vegetative stage.

### Chemical constituents

#### Photosynthetic pigments

Chlorophyll a, Chlorophyll b, and total carotenoids were extracted from 0.1 g of fresh leaves in 10 ml of 85% acetone and measured according to^38^. The homogenized samples were centrifuged at 3000 rpm, and the supernatant was up to 10 ml with acetone (85%). The absorbance was recorded at 663, 644, and 452 nm by spectrophotometer (VEB Carl Zeiss) using acetone as a blank. The concentration of the pigment fractions (chlorophyll *a*, chlorophyll *b*, and carotenoids) was accounted for as µg/ml using the following equations:

1 $${\text{Chlorophyll}}\,a = \, \left[ {\left( {{1}0.{3} \times {\text{E663}}} \right) \, {-} \, \left( {0.{918} \times {\text{E644}}} \right)} \right] \, = \, \mu {\text{g ml}}^{{ - {1}}}$$

2 $${\text{Chlorophyll}}\,b = \, [({19}.{7} \times {\text{E644}}) \, {-} \, ({3}.{87}0 \times {\text{E663}})] \, = \mu {\text{g ml}}^{{ - {1}}}$$

3 $${\text{Carotenoids}}\, = \,({4}.{2}\, \times \,{\text{E452}}) - [(0.0{264}\, \times \,{\text{chlorophyll}}a)\, + \,(0.{426}\, \times \,{\text{chlorophyll}}b)]\, = \,\mu {\text{g ml}}^{{ - {1}}}$$

The concentrations of chlorophylls and carotenoids were expressed as mg g^-1^ fresh weight (FW) of plant material.

#### Soluble sugars

Total soluble sugars (TSS) were determined in fresh leaves based on the anthrone technique according to^39^. TSS content was analyzed by reacting 0.1 mL of ethanol extract with 3 mL freshly prepared anthrone (150 mg from anthrone + 100 mL from 72% H2SO4) in a boiling water bath for 10 min and reading the cooled samples at 625 nm using a spectrophotometer (VEB Carl Zeiss). Total soluble sugar is calculated using a standard curve of glucose.

#### Soluble phenols

Total soluble phenols were determined in fresh leaves using the Folin–Ciocalteau reagent method according to^40^. One mL of the extract was added to ten drops of concentrated HCl in a boiling water bath for ten min and cooled. Followed by one ml of Folin–Ciocalteau reagent and 1.5 mL of 14% sodium carbonate were mixed. The mixture was up to 5 ml of distilled water, shaken well, and then kept in a boiling water bath for 5 min. The absorbance at 650 nm was noted, and the data were represented as mg g^−1^ FW using a pyrogallol standard curve.

#### Proline

A known weight (0.5 g) of fresh leaves was extracted in 10 ml of 3% aqueous sulfosalicylic acid. Two ml of the supernatant was mixed with 2 ml of acid ninhydrin reagent and 2 ml of glacial acetic acid, respectively. After boiling the mixture for one hour at 100 °C, it was cooled in an ice bath, and the toluene (4 ml) was added to the reaction mixture. The absorbance was recorded at 520 nm using toluene as a blank by a spectrophotometer^41^.

#### Amino-N

Amino-N in fresh leaves of flax plants was detected by the ninhydrin method according to^42^.

#### Enzymes activity

The crude enzyme extract was prepared according to^43^ to assay different enzymes related to antioxidant activity in fresh leaf samples.

#### Peroxidase (POD)

POD activity was assayed in the reaction mixture, including 0.2 ml of enzyme extract in a buffer solution containing 5.8 ml of 50 mM phosphate buffer (pH 7.0), 2.0 ml of 20 mM pyrogallol, and 2.0 ml of 20 mM H_2_O_2_. The increase in absorbance was determined at 470 nm for 60 s. One unit of enzyme activity is defined as the amount of enzyme that catalyzed the conversion of one micromole of H_2_O_2_ per min at 25 °C^44^.

#### Ascorbate peroxidase (APX)

APX activity was examined according to^45^. The reaction mixture containing potassium phosphate buffer (50 mM; pH 7.0), ascorbic acid (0.5 mM), H_2_O_2_ (1.0 mM), and 50 μl of enzyme extract in the final volume of 1 ml. The activity of APX was detected at 290 nm for 3 min.

#### Lipid peroxidation

A fresh weight (0.2 g) was extracted with 10 ml of trichloroacetic acid (1% w/v). The extract was centrifuged at 10,000 rpm for 10 min. Lipid peroxidation was measured based on the thiobarbituric acid reaction according to the assay of^46^. The supernatant (2.0 ml) was added to 4.0 ml of 0.5% thiobarbituric acid (TBA) in 20% TCA. The solution was heated at 95 °C for 30 min and then immediately cooled and centrifuged at 10000×*g* for 10 min. The absorbance was recorded at 532 and 600 nm by spectrophotometer. By subtracting the absorption value at 600 nm, the MDA content was assessed using its absorption coefficient of 155 nmol cm^−1^ and expressed as nmol g^−1^ fresh weight.

### Protein profile

Protein extract was prepared by the rapid freeze of 0.2 g of fresh leaves using liquid nitrogen then protein profiling was separated using SDS–polyacrylamide gel^47^. The molecular weights of the separated proteins were estimated against standard molecular weight markers (Marker, 15–180 kDa; Sigma, St. Louis, USA).

### Statement on guidelines

Experimental procedures and field studies on plants comply with relevant institutional, national, and international guidelines and legislation.

### Statistical analysis

Data were analyzed by using a two-way analysis of variance (ANOVA) according to^48^. The least significant differences (LSD) at a 5% level of probability were calculated to compare the means of different treatments.

## Supplementary Information

Supplementary Information.

## Data Availability

The availability of data and material data is available in a supplementary file.
