# Supplementary material for: Cysteine mitigates the effect of NaCl salt toxicity in flax (Linum usitatissimum L) plants by modulating antioxidant systems
Source: Sci Rep. 2022 Jul 5;12:11359. doi: 10.1038/s41598-022-14689-7 (PMC9256724; doi:10.1038/s41598-022-14689-7)
Supplement: Supplementary file 1 — Supplementary Information. [file 41598_2022_14689_MOESM1_ESM.pdf]

Supplementary Material for

Cysteine mitigates the effect of NaCl salt toxicity in flax (*Linum usitatissimum* L) plants by modulating antioxidant systems

Hebat-Allah A. Hussein [1,3*] and Shifaa O Alshammari[2]

[1]Botany and Microbiology Department, Faculty of Science (Girls Branch), Al Azhar University, Cairo, 11754, Egypt.

[2]Biology Department, College of Science, University of Hafr Al Batin (UHB), Hafr Al Batin, 31991, Saudi Arabia

[3]Biology Department, University College of Nairiyah, University of Hafr Al Batin (UHB), Nairiyah, 31991, Saudi Arabia

*Corresponding author: Hebat-Allah A. Hussein; hebahussein@azhar.edu.eg

Figure S1

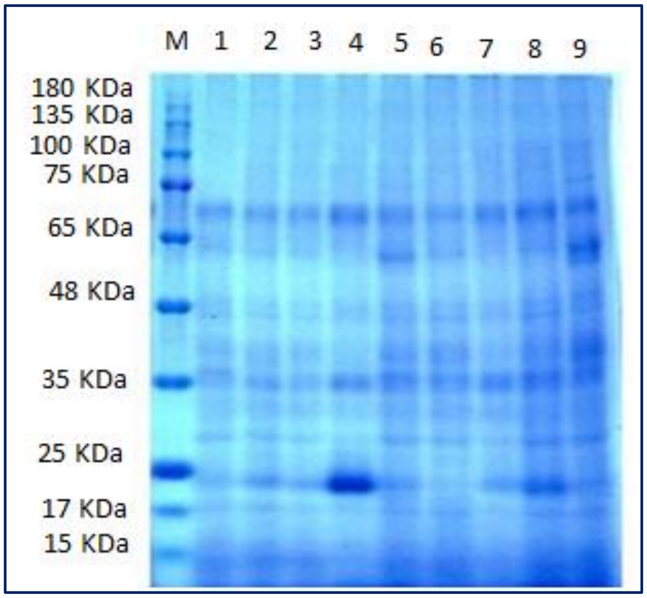

Figure S1. Effect of L- cysteine on protein profile in leaves of salt stressed flax plants. Where, M, Protein marker; 1, Control; 2, 0.8 mM Cys; 2, 1.6 mM Cys; 4, 50 mM NaCl; 5, 50 mM NaCl + 0.8 mM Cys; 6, 50 mM NaCl + 1.6 mM Cys; 7, 100 mM NaCl; 8, 100 mM NaCl + 0.8 mM Cys; 9, 100 mM NaCl + 1.6 mM Cys.

Raw Data

Treatments		Plant Height (cm) plant^{-1}			
NaCl (mM)	Cysteine (mM)	R1	R2	R3	mean
0	0	58	55	53	55.33
	0.8	56	50	53	53.00
	1.6	61	69	66	65.33
50	0	35	28	30	31.00
	0.8	56	54	58	56.00
	1.6	50	51	55	52.00
100	0	27	27	27	27.00
	0.8	43	37	41	40.33
	1.6	44	48	48	46.67

Treatments		Root Length (cm) plant^{-1}			
NaCl (mM)	Cysteine (mM)	R1	R2	R3	mean
0	0	16	18	13	15.67
	0.8	13	13	11	12.33
	1.6	13	12	13	12.67
50	0	14	14	15	14.33
	0.8	16	13	10	13.00
	1.6	11	11	11	11.00
100	0	9	8	8	8.33
	0.8	12	13	11	12.00
	1.6	18	15	12	15.00

Treatments		Shoot FW (g) plant^{-1}			
NaCl (mM)	Cysteine (mM)	R1	R2	R3	mean
0	0	2.85	2.74	2.48	2.69
	0.8	5.96	4.56	4.44	4.99
	1.6	5.61	4.70	9.39	6.57
50	0	1.91	1.86	1.79	1.85
	0.8	3.91	2.44	3.09	3.15
	1.6	6.50	6.79	6.60	6.63
100	0	1.27	1.00	1.28	1.18
	0.8	3.92	2.60	2.45	2.99
	1.6	2.97	2.38	3.82	3.05

Treatments		Shoot DW (g) plant^{-1}			
NaCl (mM)	Cysteine (mM)	R1	R2	R3	mean
0	0	0.55	0.52	0.78	0.61
	0.8	0.78	0.68	0.88	0.78
	1.6	0.88	0.98	0.92	0.93
50	0	0.44	0.46	0.44	0.45
	0.8	0.47	0.51	0.47	0.48
	1.6	0.55	0.51	0.56	0.54
100	0	0.25	0.41	0.18	0.28
	0.8	0.40	0.41	0.26	0.36
	1.6	0.39	0.32	0.23	0.31

Treatments		Root Fw (g) plant^{-1}			
NaCl (mM)	Cysteine (mM)	R1	R2	R3	mean
0	0	0.60	0.70	0.50	0.60
	0.8	1.46	1.01	1.20	1.22
	1.6	2.37	1.71	1.26	1.78
50	0	0.27	0.40	0.30	0.32
	0.8	1.03	0.91	0.78	0.91
	1.6	1.51	1.50	1.50	1.50
100	0	0.15	0.10	0.09	0.11
	0.8	0.51	0.83	0.55	0.63
	1.6	0.46	0.87	0.85	0.73

Treatments		Root Dw (g) plant^{-1}			
NaCl (mM)	Cysteine (mM)	R1	R2	R3	mean
0	0	0.110	0.040	0.100	0.08
	0.8	0.087	0.135	0.188	0.14
	1.6	0.109	0.106	0.126	0.11
50	0	0.050	0.031	0.037	0.04
	0.8	0.060	0.065	0.050	0.06
	1.6	0.062	0.093	0.080	0.08
100	0	0.020	0.029	0.026	0.03
	0.8	0.070	0.028	0.073	0.06
	1.6	0.049	0.055	0.110	0.07

Treatments		Chl a (mg g^{-1} Fw)			
NaCl (mM)	Cysteine (mM)	R1	R2	R3	mean
0	0	**1.09**	1.06	1.12	1.09
	0.8	**1.26**	1.29	1.23	1.26
	1.6	**1.29**	1.32	1.26	1.29
50	0	**0.76**	0.79	0.73	0.76
	0.8	**1.02**	1.05	0.99	1.02
	1.6	**1.15**	1.18	1.12	1.15
100	0	**0.67**	0.7	0.64	0.67
	0.8	**0.96**	0.99	0.93	0.96
	1.6	**1.18**	1.21	1.15	1.18

Treatments		Chl b (mg g^{-1} Fw)			
NaCl (mM)	Cysteine (mM)	R1	R2	R3	mean
0	0	**0.45**	0.48	0.42	0.45
	0.8	**0.53**	0.56	0.5	0.53
	1.6	**0.57**	0.6	0.54	0.57
50	0	**0.24**	0.27	0.21	0.24
	0.8	**0.37**	0.40	0.34	0.37
	1.6	**0.45**	0.48	0.42	0.45
100	0	**0.25**	0.28	0.22	0.25
	0.8	**0.32**	0.35	0.29	0.32
	1.6	**0.42**	0.45	0.39	0.42

Treatments		Carotenoids (mg g^{-1} Fw)			
NaCl (mM)	Cysteine (mM)	R1	R2	R3	mean
0	0	0.25	0.25	0.25	0.25
	0.8	0.25	0.26	0.25	0.25
	1.6	0.23	0.23	0.23	0.23
50	0	0.12	0.11	0.12	0.12
	0.8	0.14	0.16	0.15	0.15
	1.6	0.21	0.2	0.21	0.21
100	0	0.11	0.12	0.11	0.11
	0.8	0.12	0.14	0.13	0.13
	1.6	0.22	0.23	0.23	0.23

Treatments		TSS (mg g^{-1} Fw)			
NaCl (mM)	Cysteine (mM)	R1	R2	R3	mean
0	0	4.21	3.81	4.01	4.01
	0.8	3.56	3.65	3.60	3.60
	1.6	3.00	3.24	3.12	3.12
50	0	2.84	2.92	2.88	2.88
	0.8	2.84	2.75	2.79	2.79
	1.6	2.43	2.51	2.47	2.47
100	0	3.00	3.32	3.16	3.16
	0.8	2.59	2.51	2.55	2.55
	1.6	1.86	2.43	2.15	2.15

Treatments		Phenols (mg g^{-1} Fw)			
NaCl (mM)	Cysteine (mM)	R1	R2	R3	mean
0	0	2.19	2.28	2.23	2.23
	0.8	1.87	1.87	1.87	1.87
	1.6	1.70	1.70	1.70	1.70
50	0	1.70	1.61	1.66	1.66
	0.8	2.08	2.11	2.10	2.10
	1.6	1.75	1.75	1.75	1.75
100	0	1.44	1.34	1.39	1.39
	0.8	1.73	1.84	1.79	1.79
	1.6	1.52	1.61	1.57	1.57

Treatments		Proline (µmol g^{-1} FW)			
NaCl (mM)	Cysteine (mM)	R1	R2	R3	mean
0	0	2.29	2.37	2.33	2.33
	0.8	1.26	1.69	1.47	1.47
	1.6	1.34	1.57	1.45	1.45
50	0	2.10	3.20	2.65	2.65
	0.8	0.96	0.79	0.88	0.88
	1.6	0.72	0.84	0.78	0.78
100	0	3.86	2.01	2.94	2.94
	0.8	1.05	1.03	1.04	1.04
	1.6	0.98	0.83	0.90	0.90

Treatments		Amino –N (mg g^{-1} FW)			
NaCl (mM)	Cysteine (mM)	R1	R2	R3	mean
0	0	2.99	2.98	3.00	2.99
	0.8	2.82	2.85	2.87	2.85
	1.6	2.90	2.93	2.88	2.90
50	0	2.42	2.35	2.36	2.38
	0.8	2.80	2.70	2.65	2.72
	1.6	2.75	2.80	2.70	2.75
100	0	2.07	2.00	2.03	2.03
	0.8	2.55	2.59	2.65	2.60
	1.6	2.66	2.70	2.72	2.69

Treatments		Peroxidase (U h^{-1} g^{-1} FW)			
NaCl (mM)	Cysteine (mM)	R1	R2	R3	mean
0	0	583.2	583.2	583.2	583.20
	0.8	100.8	100.0	100.4	100.40
	1.6	360.0	300.0	360.0	340.00
50	0	482.4	648.0	565.2	565.20
	0.8	72.0	72.0	72.0	72.00
	1.6	324.0	300.0	312.0	312.00
100	0	115.2	169.2	142.2	142.20
	0.8	28.8	32.4	30.6	30.60
	1.6	36.0	54.0	45.0	45.00

Treatments		Ascorbate peroxidase (U h^{-1} g^{-1} FW)			
NaCl (mM)	Cysteine (mM)	R1	R2	R3	mean
0	0	171.6	204.0	187.8	187.80
	0.8	72.8	88.8	80.8	80.80
	1.6	42.4	63.2	52.8	52.80
50	0	130.8	127.2	129.0	129.00
	0.8	81.2	76.0	78.6	78.60
	1.6	40.0	60.8	50.4	50.40
100	0	118.0	96.8	107.4	107.40
	0.8	88.0	62.0	75.0	75.00
	1.6	40.8	39.6	40.2	40.20

Treatments		MDA (µmol g^{-1} FW)			
NaCl (mM)	Cysteine (mM)	R1	R2	R3	mean
0	0	3.87	4.19	4.03	3.87
	0.8	2.26	2.58	2.42	2.26
	1.6	3.27	3.23	3.25	3.27
50	0	1.94	6.77	4.35	1.94
	0.8	2.21	2.44	2.33	2.21
	1.6	2.94	3.55	3.24	2.94
100	0	6.23	7.10	6.66	6.23
	0.8	2.97	3.16	3.06	2.97
	1.6	5.48	5.48	5.48	5.48
